# Assessing positive and negative valence systems to refine animal models of bipolar disorders: the example of GBR 12909-induced manic phenotype

**DOI:** 10.1038/s41598-022-10965-8

**Published:** 2022-05-05

**Authors:** Mathilde Bigot, Eleonore Vicq, Pierre-Marie Lledo, Mariana Alonso, Chantal Henry

**Affiliations:** 1 Institut Pasteur, Université Paris Cité, CNRS UMR 3571, Perception and Memory Unit, 25 rue du Docteur Roux, F-75015 Paris, France; 2 Sorbonne Université, Collège doctoral, F-75005 Paris, France; 3 Université de Paris Cité, Paris, France; 4 Department of Psychiatry, Service Hospitalo-Universitaire, GHU Paris Psychiatrie & Neurosciences, Paris, France

**Keywords:** Bipolar disorder, Diseases of the nervous system, Emotion, Gustatory system, Olfactory system

## Abstract

Bipolar disorders are defined by recurrences of depressive and manic episodes. The pathophysiology is still unknown, and translating clinical symptoms into behaviors explorable in animal models is challenging. Animal models of bipolar disorder do not exist because cyclicity of the disease is impossible to mimic, and it is therefore necessary to study mania and depression models separately. Beyond mood, emotional biases differentiate bipolar states in humans. Mania is associated with positive biases, e.g. emotional stimuli become more rewarding and less aversive, and the opposite for depression. We propose to assess behavioral hedonic responses to innately appetitive and aversive olfactory and gustatory cues in mice as proxies for the assigned emotional valence. A mania model is therefore supposed to exhibit positive hedonic bias. Using the GBR 12909 mania model, we observed the classical hyperactivity phenotype, along with low depressive-like but high anxiety-like behaviors. Unexpectedly, GBR 12909-treated mice exhibited strong negative hedonic biases. Consequently, the GBR 12909 model of mania might not be appropriate for studying emotional disturbances associated with mania states. We propose olfactory and gustatory preference tests as crucial assessment for positive and negative valence biases, necessary for precisely characterizing animal models of bipolar disorders.

## Introduction

Bipolar disorders (BD) are defined by alternating phases of depression, mania or mixed exaltation, separated by periods of remission. Depressive and manic episodes are characterized by opposite symptoms (sadness/expansible mood, fatigue/increased energy, psychomotor retardation/agitation, etc*.*) and mixed states by a mixture of both depressive and manic symptoms. Due to subjectivity of diagnosis and reporting, these clinical criteria are difficult to transpose to animal models^1^. Such obstacles hinder the transfer of the immense progress in pre-clinical neuroscience made over the last 2 decades about neural mechanisms involved in major brain functions to the psychiatric field.

To overcome this issue, we need to translate the clinical criteria into domains or basic function alterations that are measurable both in humans and animals to be accessible for neurobiological multilevel analysis, following the RDoC (Research Domain Criteria) initiative^2–4^. Emotional processing, which belongs to the positive and negative valence domains, is an essential domain affected in BD. However, this field of research has long been neglected due to the lack of clear definition of emotions that is suitable across species. The current consensus stipulates that emotions are brief physiological and behavioral responses to stimuli described by several dimensions, including valence^5,6^. Moreover, emotion expressions are thought as windows into the internal affective state of an individual across species from insect to humans^6^. Based on this operational formalization, emotional processes can be explored both in clinical and pre-clinical studies. For instance, approach or avoidance behaviors are quantifiable motor readouts indicating the animal assigned valence of the presented stimuli and expressing an indirect measure of the triggered emotion^6,7^.

We recently proposed an emotion-based model for bipolar disorders, contrasting with the current diagnostic frame built on mood^8^. According to our model developed from clinical data, many symptoms of manic and depressive states are linked to emotional biases, e.g. misattribution of the hedonic value or valence of stimuli. In manic patients, positive emotional biases lead to increased search of pleasure and risk-taking behaviors by decreasing the perception of danger^8,9^. Conversely, depressive states are characterized by emotional negative biases accounting for loss of pleasure^8,10,11^. It is therefore crucial to study emotional biases in animal models of BD to better understand the pathophysiology of alterations on this key dimension**.** Importantly, thanks to recent techniques allowing the manipulation of specific neuronal networks, neuroscience has already made considerable progress in the knowledge of emotional valence neural processing^12^.

Animal models for BD are restricted to mania models because the cyclicity of the disease is almost impossible to reproduce^3^. These models have relied on pharmacological, genetic or environmental manipulations. Because a dysregulated dopaminergic system seems implicated in the etiopathology of the manic state, and because psychostimulants in humans induce manic symptoms, the injection of amphetamine is a common way to create a manic-like state in animals^13–15^. However, this drug is also used for modeling other diseases such as schizophrenia, ADHD and drug abuse, and therefore is not specific for BD^14,15^. Consequently, amphetamine induces not only hyperlocomotion, but also addiction and hallucinations, generating several confounding factors. Another model has been developed based on a very specific dopamine reuptake inhibitor, GBR 12909^16^. Human studies reported that polymorphisms of the gene coding for the dopamine transporter are associated with BD^17,18^ and reduced striatal dopamine transporter levels have been observed in BD patients^19^. Like classical psychostimulants, GBR 12909 induces significantly increased locomotor activity and a hyper-exploratory profile such as seen in BD patients^16^. Moreover, the effects on locomotion are reversed by lithium, valproate or aripiprazole^20^.

Therefore, based on its apparent correct face, construct and predictive validity criteria, we chose the GBR 12909 mania model to assess hedonic biases in response to olfactory and gustatory preference tests. Locomotion, anxiety and depressive-like behaviors were measured to complete the phenotype evaluation. We initially hypothetized GBR 12909 to trigger positive hedonic biases in response to innately attractive or aversive odor and taste cues.

## Results

### GBR administration triggers hyperlocomotion, anxiety-like behaviors and combativeness

**Figure 1:**
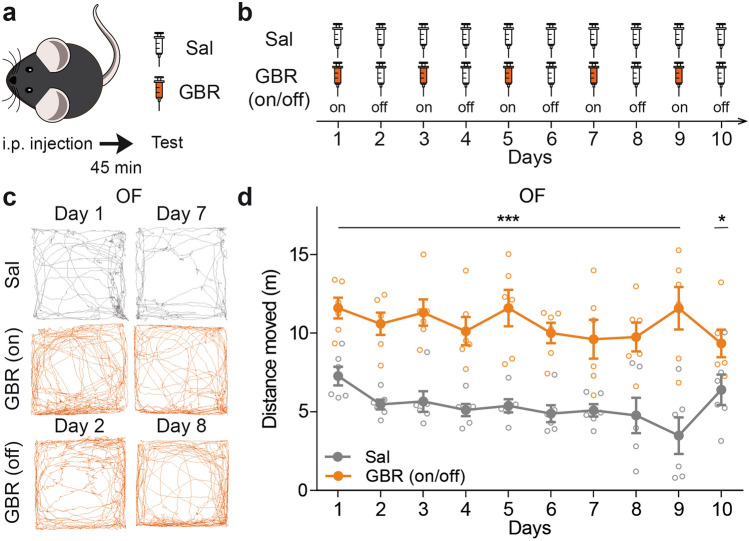
GBR administration induces strong hyperlocomotion. (**a**) Either GBR 12909 (GBR), 16 mg/kg, or saline (Sal) was injected intra-peritoneally (i.p.) 45 min before behavioral testing. (**b**) GBR was administered every other day (“on”), alterned with saline (“off”) during 10 days to obtain a sub-chronic model for mania. (**c**) Representative animal track for the 5 first minutes of the Open field test (OF). One same animal is showed for Sal group on days 1 and 7, and for the GBR group on days 1, 7 (“on”) and days 2 and 8 (“off”). (**d**) OF revealed sustained hyperlocomotion in the GBR (on/off)-treated mice compared with the Sal-treated mice over 10 days (two-way repeated measures ANOVA, group: F_(1,10)_ = 45.42, ***p < 0.001, days: F_(9,90)_ = 1.98, p = 0.050, interaction: F_(9,90)_ = 1.91, p = 0.060, n = 6). Data are shown as mean ± SEM and individual data points.

To assess hedonic biases, we needed a stable model for mania across multiple days. We injected GBR 12909 (GBR) every 2 days for 10 days (Fig. 1a,b), alterning with saline (Sal) administration. Using the Open field test (OF), 45 min after either Sal or GBR administration (“on” days), GBR-injected mice showed a significant increase in locomotor activity relative to Sal-injected mice (Fig. 1c,d). We observed that this regime of GBR administration maintained high levels of locomotion compared to Sal-treated mice during the whole 10 days of the experiment (Fig. 1c,d). In particular, the typical hyperlocomotion observed for the “on” days was sustained to the “off” days. In contrast, daily GBR injections might have induced tolerance to the drug (Supplementary Fig. 1). Our results showed that injecting GBR every 2 days generates a robust and stable hyperlocomotion distinctive of mania models.

**Figure 2:**
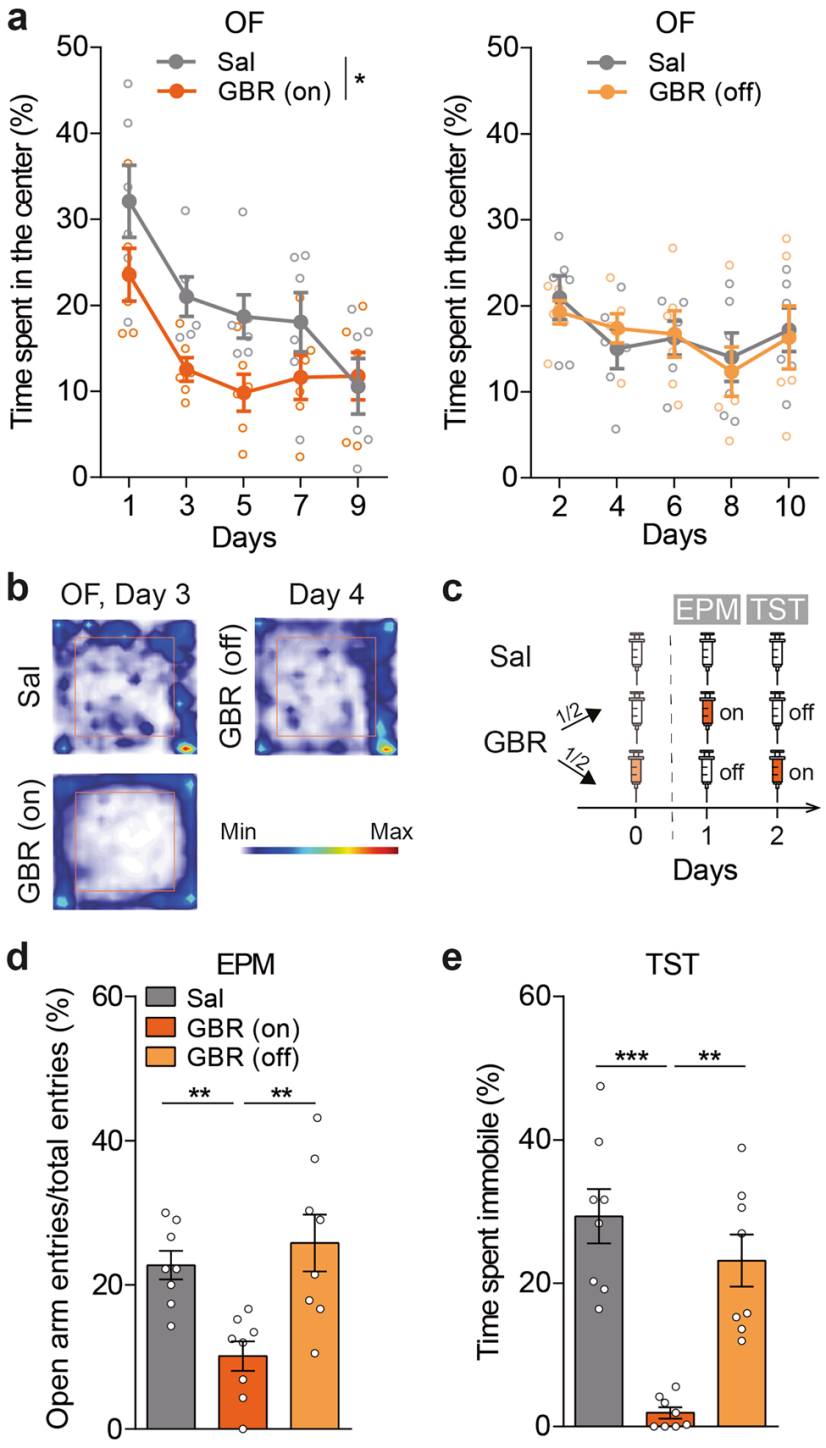
Anxiety-like behaviors co-existed with anti-depressant-like phenotype in GBR-treated mice. (**a**) GBR-treated mice spent less time in the OF center compared to Sal-treated mice during the “on” days (left, Two-way repeated measures ANOVA, group: F_(1,10)_ = 5.61, *p = 0.039, days: F_(4,40)_ = 13.89, p < 0.001, interaction : F_(4,40)_ = 1.54, p = 0.209, n = 6), but not during the “off” days (right, two-way repeated measures ANOVA, group: F_(1,10)_ = 0.01, p = 0.911, days: F_(4,40)_ = 3.07, p = 0.027, interaction : F_(4,40)_ = 0.38, p = 0.821, n = 6). (**b**) Representative heatmaps of the mean position in the OF of Sal and GBR (on) animals (day 3) and GBR (off) animal (day 4). The orange squares delimit the center of the arena. (**c**) Scheme of the injection protocol to behaviorally test GBR (on) and (off) animals in the Elevated Plus Maze test (EPM) and Tail Suspension Test (TST). (**d**) In the EPM, GBR (on) mice showed less entries in the open arms/total entries, relative to Sal and GBR (off) mice (one-way ANOVA, F_(2,21)_ = 8.78, p = 0.002 followed by Holm–Sidak post-hoc test, **p < 0.01, n = 8). (**e**) GBR (on) mice spent less time immobile in the TST, compared to Sal and GBR (off) mice (Kruskal–Wallis test, H = 16.24, p < 0.001, Dunn post-hoc analysis, **p < 0.01, ***p < 0.001, n = 8). Data are shown as mean ± SEM and individual data points.

Remarkably, GBR-treated mice exhibited lower center exploration in the OF than Sal-treated mice, indicating anxiogenic effects of the drug (Fig. 2a left, b). This anxiety-like phenotype was present only on “on” days and not on “off” days (Fig. 2a right, b). To confirm this observation, we measured the behavior in a classical anxiety paradigm, the Elevated Plus Maze test (EPM). We split the GBR-treated mice into two groups, GBR (on) injected on the day of test and the GBR (off) injected the day before (Fig. 2c). Consistently with the OF, the GBR (on) mice showed a reduced percentage of entries into the open arms relative to Sal and to GBR (off) mice (Fig. 2d). This anxiety-like phenotype was not accompanied by a depressive-like state of the animals. Indeed, GBR administration 45 min before Tail Suspension Test (TST) almost completely suppressed immobility, whereas the administration the day before did not (Fig. 2e). Therefore, GBR administration in mice triggered at short-term the major components of mania including hyperlocomotion and an increased combativeness phenotype, although these features co-existed with anxiety-like behaviors.

### Strong negative olfactory hedonic bias in GBR-treated mice

**Figure 3:**
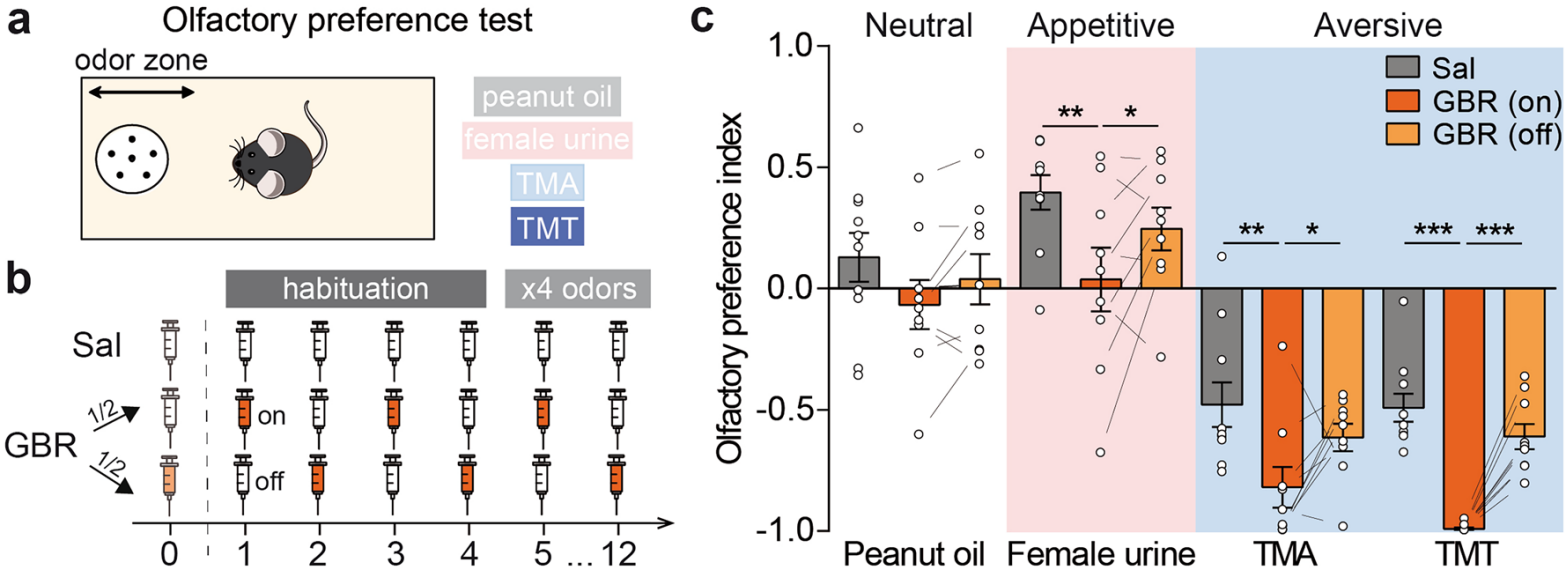
The GBR mice model exhibit acute negative hedonic biases in olfactory preference test. (**a**) Scheme of the olfactory preference test protocol. After 4 days of habituation, each odor was presented on two consecutive days. Peanut oil and female urine are used as innate neutral and attractive odors, respectively. TMA (trimethylamine) and TMT (trimethylthiazoline) are synthetic predator odors triggering innate aversion. (**b**) Timeline of the injections. GBR-treated mice were divided into two groups, altenatively injected each day with either Sal or GBR. (**c**) The olfactory preference index, calculated with time spent in the odor zone during odor presentation relative to habituation is a proxy for the valence attributed to each odor by the mice. In our experiments, the peanut oil was neutral in Sal-treated mice (Student test vs a theoretical value 0, t = 1.265, df = 9, p = 0.238, n = 10; see also Supplementary Table 1). GBR (on) mice exhibited a lower olfactory preference index than Sal mice for all non-neutral odors, meaning a decreased attractiveness of the female urine odor, and an increased aversiveness of the TMA and TMT, which was not present during GBR (off) (Sal vs GBR (on): two-way repeated measures ANOVA, group: F_(1,17)_ = 15.94, p < 0.001, Odor: F_(3,51)_ = 90.93, p < 0.001, interaction: F_(3,51)_ = 1.53, p = 0.217 followed by Holm–Sidak post-hoc test, *p < 0.05, ***p < 0.001, n = 9–10; Sal vs GBR (off): two-way repeated measures ANOVA, group: F_(1,17)_ = 3.53, p = 0.078, odor: F_(3,51)_ = 68.52, p < 0.001, interaction: F_(3,51)_ = 0.06, p = 0.981, n = 9–10). The “on” and “off” days were significantly different in GBR-treated mice upon presentation of female urine, TMA and TMT (GBR (on) vs GBR (off): two-way repeated measures ANOVA, on/off: F_(1,8)_ = 14.49, p = 0.005, odor: F_(3,24)_ = 52.64, p < 0.001, interaction: F_(3,24)_ = 2.27, p = 0.107 followed by Holm–Sidak post-hoc test, *p < 0.05, ***p < 0.001, n = 10). Data are shown as mean ± SEM and individual data points.

We next performed an olfactory preference test measuring innate behavioral hedonic responses to both appetitive and aversive odor stimuli as an indirect way to assess emotional states biases (Fig. 3a, see “Materials and methods”). GBR-treated mice were divided into two groups so that in each day of odor exposure both GBR (on) and GBR (off) mice were tested (Fig. 3b). The exposure to peanut oil resulted in a preference index no different from zero in Sal-treated mice, indicating this stimulus was neutral in our experimental conditions (Fig. 3c, Supplementary Table 1). However, the stimulation with female urine induced an approach response in Sal-treated mice, as reflected by the positive preference index (Supplementary Table 1). In contrast, TMA as well as TMT were aversive, as demonstrated by negative olfactory indexes (Supplementary Table 1). Interestingly, GBR (on) mice exhibited a lower olfactory preference index than Sal mice for all non-neutral odors, causing decreased attractiveness to female urine and increased aversiveness to TMA and TMT (Fig. 3c). On the “off” days, the index changed compared to the “on” days, becoming no statistically different from the Sal-treated mice, despite a trend for the group effect. Aversive odors usually decreases locomotor activity in control mice^21^. In GBR-treated mice, it is worth noting that the aversiveness of TMT was sufficient to prevent the hyperlocomotion phenotype of GBR (on) but not of GBR (off) mice (Supplementary Fig. 2). In conclusion, the GBR (on) mice exhibited global negative hedonic bias in the olfactory preference test and, importantly, this effect was specific for odors triggering approach or avoidance (e.g. not neutral) as it was not present for peanut oil or during habituation (Supplementary Fig. 2).

**Figure 4:**
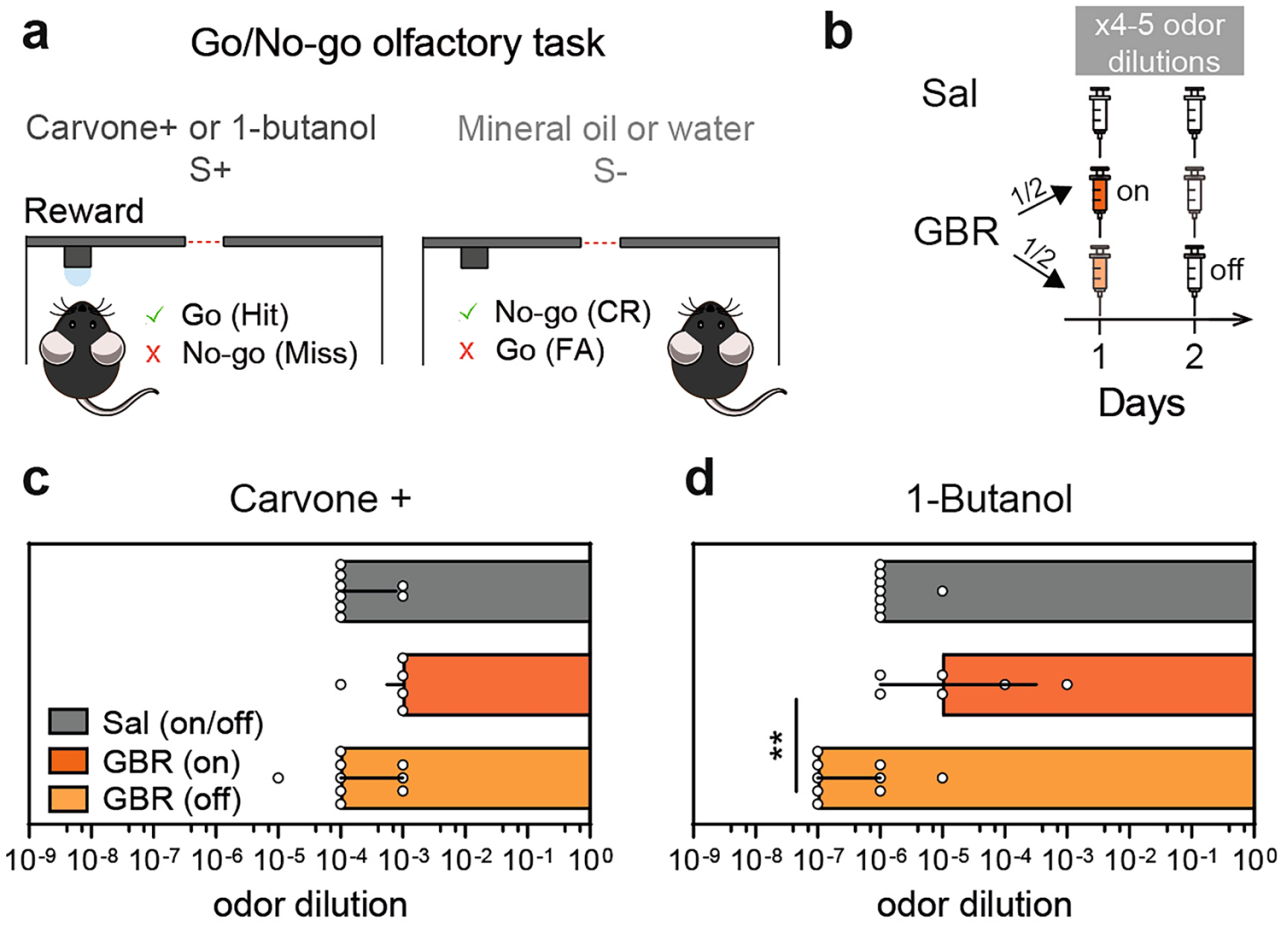
GBR administration does not alter odor detection threshold. (**a**) Schematic of the Go/no-go learning task. In response to S+, licking the water port triggered water delivery. This constitutes a correct response (Go, a hit), whereas not going to lick is considered an error (No-go, a miss). In response to S−, trained mice refrained from licking, thus constituting the correct response (No-go, correct rejection, CR), whereas going to lick constitutes an error (Go, false alarm, FA). (**b**) Experimental timeline and injection protocol. All mice were first trained in a detection task without any treatment (carvone+ (10^–2^) *vs* mineral oil; 1-butanol (10^–3^) *vs* water, data not shown). Performances were similar for all groups (Mixed-effect model, carvone+, group: F_(1.772,14.18)_ = 0.34, p = 0.691, n = 5–9; 1-butanol, group: F_(1.429,11.44)_ = 0.17, p = 0.778 n = 8–9). (**c**,**d**) Detection threshold concentration were determined in mice injected with Sal or GBR every other day. Sal and GRB-injected mice were divided in two sub-groups, and the animals were tested 45 min (“on”) or 24 h (“off”) after injections. Sal (on) and (off) showed identical performances and were pooled together (Mann–Whitney test, carvone+, p = 0.429, n = 4; 1-butanol, p > 0.999, n = 4). Odorant concentration (v/v) are given as the dilutions used in the olfactometer. GBR administration does not alter odor detection threshold for both carvone+ (**c**) and 1-butanol (**d**) with respect to the Sal group**.** A significant difference was found for 1-butanol detection threshold between GBR (on) and (off) (Kruskal–Wallis test, carvone+: p = 0.165; 1-butanol: p = 0.008, followed by Dunn’s test: **p < 0.01). Data are shown as median with interquartile range and individual data points.

We next checked whether the observed hedonic bias could be explain by olfactory modifications following GBR administration. To do so, we evaluated odor detection threshold using a go/no-go operant conditioning paradigm. In this test, water-deprived mice were trained to discriminate between a pair of stimuli: a reinforced odor associated with a water reward (positive stimulus: S+; Fig. 4a, left) and an unreinforced odor (negative stimulus: S−; Fig. 4a, right). Mice were tested in two independent tasks to recognize carvone+ or 1-butanol as the stimulus rewarded, and their solvant (mineral oil and water, respectively) as the non-rewarded stimulus. All mice were first trained to detect an odor at a high concentration without any treatment (10^–2^ v/v for carvone+ and 10^–3^ v/v for 1-butanol), showing similar performances between groups (data not shown; Mixed-effect model, carvone+, Group : F_(1.772, 14.18)_ = 0.34, p = 0.691, n = 5–9; 1-butanol, Group : F_(1.429, 11.44)_ = 0.17, p = 0.778 n = 8–9). Then, detection threshold concentrations were determined by presenting successively descending decimal concentrations of carvone+ and 1-butanol. Sal and GBR-injected mice were divided in two sub-groups, and the animals were tested 45 min (“on”) or 24 h (“off”) after injections (Fig. 4b). We found that relative to Sal mice, neither GBR (on) nor GBR (off) groups had deficits in detection threshold for carvone+ (Fig. 4c) or 1-butanol (Fig. 4d). The only statistically significant difference was found for butanol detection threshold between GBR (on) and (off) using very low odor concentrations. In addition, the detection and movement times between groups remained unchanged, supporting the notion that odor investigation did not vary with GBR administration (Supplementary Fig. 3). Overall, our results showed that GBR administration does not alter odor detection threshold in a way that could influence the response to odors in our olfactory preference test.

### Negative gustatory hedonic bias in GBR-treated mice

**Figure 5:**
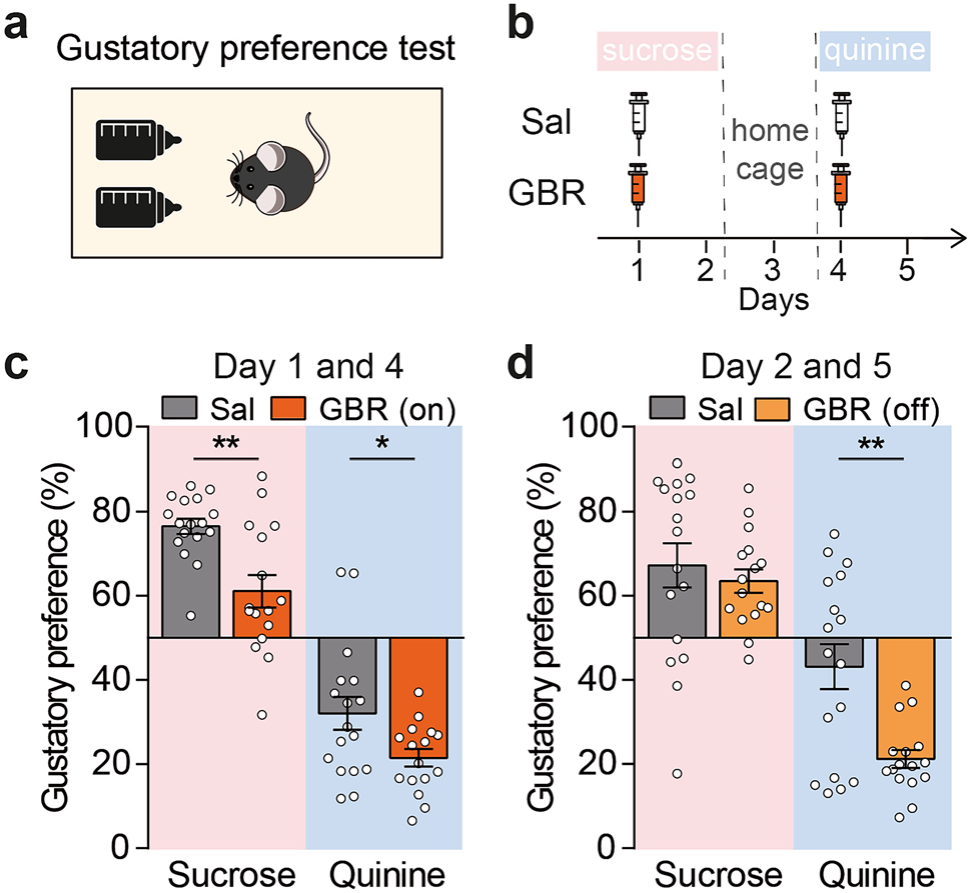
GBR-treated mice show negative hedonic biases in gustatory preference test. (**a**) Scheme of the gustatory preference test. The test occurred in a standard housing cage, with two bottles containing either water or the tastant solution. After one day of habituation with two water bottles, each tastant solution was presented *vs* water for 48 h. (**b**) Timeline of the injections protocol for the gustatory preference test. Mice were injected at the beginning of the test. (**c**,**d**) Sucrose is an appetitive tastant in Sal-treated mice both during the first and the last 24 h of test, whereas quinine is aversive for these mice only during the first 24 h (Student or Wilcoxon test vs a theoretical value 50, see also Supplementary Table 2). GBR (on) mice showed a lower preference for both sucrose and quinine solution compared with Sal mice (two-way repeated measures ANOVA, group: F_(1,31)_ = 17.09, p < 0.001, tastant: F_(1,31)_ = 197.8, p < 0.001, interaction: F_(1,31)_ = 0.65, p = 0.426, Holm–Sidak post-hoc test, *p < 0.05, **p < 0.01, n = 16–17). Contrary, GBR (off) mice only exhibited a stronger aversion for quinine, relative to Sal mice for which quinine is not aversive, and no difference for sucrose preference (two-way repeated measures ANOVA, group : F_(1,31)_ = 8.13, p = 0.008, tastant: F_(1,31)_ = 70.69, p < 0.001, interaction: F_(1,31)_ = 5.29, p = 0.028 followed by Holm–Sidak post-hoc analysis, **p < 0.01, n = 16–17). Data are shown as mean ± SEM and individual data points.

To go further, we evaluated if the olfactory negative hedonic bias observed in the GBR (on) mice could also be detected in another sensory modality by evaluating gustatory preference (Fig. 5a, see “Materials and methods”). Sucrose was used as an appetitive stimuli and quinine as the aversive one which were presented for 48 h along with water in a two-bottle choice test. Mice were injected with either Sal or GBR at the beginning of the 48 h (Fig. 5b). Sucrose was indeed attractive for Sal-treated mice in both the 0–24 h and the 24–48 h periods (day 1 and 2, Fig. 5c,d, Supplementary Table 2), as reflected by gustatory preference superior to 50%. On the contrary, quinine was aversive in these mice, as they exhibited a gustatory preference lower than 50%, but only during the first 24 h (day 4 and 5, Fig. 5c,d, Supplementary Table 2). Consistently with what we observed in the olfactory preference test, GBR (on) mice expressed lower appetite for sucrose and increased aversion for quinine than Sal mice during the 0–24 h period (day 1 and 4, Fig. 5c). Whereas quinine was no longer aversive during the last 24 h in Sal-treated mice, GBR-treated mice still avoided this tastant causing Sal and GBR (off) mice had different gustatory preference for quinine (day 2 and 5, Fig. 5d). However, no differences were observed between the Sal and the GBR (off) mice in their preference for sucrose. We noticed that Sal-treated mice drank more than GBR-treated mice during all the gustatory preference tests, except during the 24–48 h period of the quinine presentation (day 5, Supplementary Fig. 4). Altogether, the olfactory and gustatory preference tests brought to light the negative hedonic olfactory and gustatory biases provoked by the acute administration of GBR in mice.

## Discussion

We used olfactory and gustatory hedonic responses in a pharmacological mania model to study emotional biases, an essential, yet overlooked dimension of mood state in humans. For that purpose, we evaluated behavioral responses to olfactory and gustatory stimuli with both positive and negative innate valences in rodents. To our surprise, we found, apart from the classical features of mania models (hyperlocomotion and anti-depressant-like phenotype), very strong negative hedonic biases in both olfactory and gustatory preference tests shortly following GBR administration. Although hyperlocomotion is sustained, emotional valence system alterations disappeared at 24 h, suggesting a time- or dose-dependent effect. Different sensitivity to the GBR dopaminergic action of the distinct brain regions implied either in locomotion or in emotion systems could explain these results.

Therefore, multi-dimensional behavioral assessment revealed that the GBR model, rather than mimicking mania, exhibits mixed-like features. During mixed states in humans, mood must be both exalted and sad simultaneously or within a very short period. Beyond the fact that mood is a persistent and slow-moving feeling and may not be appropriate to describe mixed states, the co-occurrence of manic and depressive symptoms has never been modeled in animals, to our knowledge. Following acute GBR administration, characteristics of manic-like and depressive-like states co-existed, namely an increased locomotor activity classically observed in mania models, and negative emotional biases expected to occur in models of depression. This particular phenotype may resemble to a human manic or hypomanic episode with mixed features as defined by the DSM-V, combining the increased energy (hyperlocomotion) of mania with dysphoria and/or anhedonia (negative emotional biases) of depression^22^.

First, this model clearly showed increased locomotor activity, a parameter well-described in the literature and often considered as the gold standard of mania models^16,20,23–25^. Compared to previous studies mainly studying the locomotor modifications after one single injection, we observed a stable induction of locomotor hyperactivity by administrating GBR every other day. Similar results were previously found by Queiroz et al. when they compared animals injected 2 h vs 24 h before^24^. In contrast, daily injections might have caused tolerance to the drug in our settings. Unlike these results, de Queiroz et al. did not observed any difference in locomotor activity between the 1st and 14th day of traitment with daily GBR injections^26^. This difference could be due to the GBR doses (16 vs 10 mg/kg), test conditions (day vs night) and/or the mice strains (C57Bl/6NTac vs Swiss).

We found that GBR administration increases anxiety-like behavior, at least in the “on” condition, as it was previously described for amphetamine treatment^27^. These data go against several reports showing low-anxiety-like phenotypes in different genetic models of mania^3^. It is important to stress that anxiety-like measurements were not always clearly distinguished from risk-preference behaviors, sometimes measured by the Iowa Gambling test^25^. Nonetheless, anxiety is not a clinical criteria for mania diagnosis, and would rather be associated with mixed features.

We also measured strong increased combativeness effects on the TST after acute GBR administration. A similar effect was already reported in the literature using the forced swimming test^28^. Interestingly, this effect disappeared on the day after injection, whereas locomotor hyperactivity persisted.

In contrast with its anti-depressive-like properties in the TST, acute GBR induced negative hedonic biases. Indeed, testing olfactory and gustatory preference uncovered a reduced attractivity to pleasant stimuli while unpleasant stimuli became more aversive. These hedonic biases observed with olfactory cues could not be explain by alteration of the odor detection threshold, since there was no significant difference between Sal and GBR (on) mice. All other parameters analyzed in our study support that olfactory function is not notably altered after GBR administration. Moreover, odor concentrations used in the olfactory preference test were extremely high. A previous work used a female urine sniffing test to measure reward-seeking behavior in the GluR6-KO model for mania^29^. They observed increased female urine exploration in these mice compared to control mice, as we initially expected in the GBR model. However, Kamdar et al.^30^, showed that acute injections of GBR resulted in a dose-dependent reduction in sugar water intake, consistently with our results. When examining studies involving sucrose preference in mania models, it appears that only genetic models show increased sucrose consumption^31–34^. It would be interesting to evaluate hedonic biases in different kinds of mania models to decipher if the emotional dimension is altered in a positive way only in genetic models, and to state on the presence of mixed-like features in the other kinds of pharmacological or environmental models. Another study examinated GBR-treated mice behavioral responses to damp cloth impregnated with domestic female cat fur, and concluded with increased risk-preference through increased exploration of this predator stimuli^26^. However, the olfactory stimulus used was extremely uncontrolled, and further experiments are necessary to clarify these discrepancies with our results. Finally, our protocol only evaluated behavioral hedonic responses to well-defined innate positive and negative cues that do not require previous experience to avoid any confounding effects due to potential impaired cognitive processes in the GBR model (e.g. attention or memory). Indeed, another test developed by Robinson et al.^35^ to evaluate affective biases resulting from acute intake of pro-depressant or antidepressant drugs relies on modulation of associative emotional memory.

Contrary to other classes of dopamine transporter inhibitors also acting as dopamine agonists, i.e. the amphetamines and cocaine, the GBR dopaminergic activation is lower in vivo. Indeed, GBR 12909 binds to the dopamine transporter approximately 50 times more strongly than cocaine^36^, but simultaneously inhibits the release of dopamine^37^. These combined effects only slightly elevate dopamine levels, which may dysregulate valence systems producing negative emotional biases while increasing locomotor activity, due to specific dopamine actions on distinct brain regions. Such differential action of dopamine on locomotor and emotional brain systems might explain the mixed-like phenotype observed in GBR-treated mice. Nevertheless, mixed states in human are poorly understood and their underlying mechanisms completely unknown. In this view, developing animal models of mixed states is of particular interest.

Our results demonstrate the importance of multi-dimensional assessment of animal models. In the DSM-5^22^, the two main criteria to define a manic state are elevated and expansive mood with increased activity and energy. It is therefore particularly important to have translational evaluations of these two elements. While the second criterion has largely been retained to define mania models, based on the increased motor and exploratory activity of animals, the mood main criterion has to date not found an equivalent measure applicable to animals. Indeed, mood is a subjective symptom peculiar to human nature. However, as proposed in our emotional-based model for bipolar patients, emotional biases can be explored both in humans and animals as a proxy for mood^8^. Our data support the notion that GBR-treated mice exhibit a mixed-like state in which manic and depressive symptoms occur simultaneously, unlike classical mania models. We therefore propose that emotional states bias assessment via hedonic responses is a reliable and essential method to model and characterize different BD states. The attribution of emotional valence is underpinned by mechanisms common to different sensory modalities and is well-conserved across species^38–40^. Studying hedonic biases in animal models of BD could unravel the underlying biological mechanisms involved in mood alteration.

Animal models are crucial to understand the physiopathology of a disease, but also for the development of targeted pharmacological treatments. Our results underscore the need to test in pre-clinical phases how potential anti-depressant drugs restore emotional biases. Indeed, human models propose that the direct effect of successful anti-depressant treatment may be to improve emotional negative biases^11^. GBR was first suggested as a new anti-depressant drug, based on classical pre-clinical depression tests^28,41^, although we uncovered the fact that it induces strong negative biases.

To conclude, we propose to translate the mood criteria of BD into emotional biases to more closely link neurobiological findings in rodents with human studies. The occurence of hedonic biases in animal models opens up great prospects for understanding the neural substrates of dysfunctional mood states, and most importantly provides an opportunity to discover new antidepressant and mood stabilizer drugs capable of restoring them.

## Materials and methods

### Animals

All animal care and experimental procedures followed national and European (2010/63/EU) guidelines and were approved by the French Ministry of Research (APAFiS: #16380-2018080217358599_v1). Data reporting is in compliance with ARRIVE guidelines. C57BL/6N male mice (12–16 weeks old) purchased from Taconic Farms (Denmark) were used for all behavioral tests (n = 74). We used the minimum number of animals per group, estimated from our previous knowledge in performing the same type of experiments and using as primary outcome measure the olfactory preference test (expected difference between the means of the groups: 40%, estimated standard deviation of each group: 35%, alpha risk = 0.05, beta risk = 0.8). Mice were socially housed, 4–6 per cage, and maintained under standard housing conditions (23 ± 1 °C; humidity 40%) with a 14/10 h light/dark cycle (lights on from 6:30 a.m. to 9:30 p.m.) and food and water ad libitum, except for some behavioral experiments.

### Drugs

GBR 12909 dihydrochloride (16 mg/kg, Sigma-Aldrich, Germany) was prepared in sterile saline solution for a final injection volume of 0.1 ml/10 g body weight, and dissolved after ~ 45 min heating at 40–60 °C. The animals received freshly made up solutions through intra-peritoneal injections between 0.5 and 1 h before behavioral tests. Animal were randomly allocated to the experimental groups between cages for the Sal vs GBR treatments. To minimize potential confounding factors cages were located next to in the animal house (same room and rack) and behavioural tests were performed simultaneously in both groups.

### Behavioral assessment

Before starting behavioral tests, mice were handled ~ 30 s at least twice a day for 3 days to habituate to the experimenter. All behavioral tests were conducted on separate days during the period of light (10 a.m.–7 p.m.).

#### Open-field

Animals were placed in Plexiglas containers (43 × 43 cm) and their behavior was recorded by a video camera during 20 min. A tracking system (Noldus Ethovision 3.0, Netherlands) was used to map the center and to measure the time spent in this zone and the total distance moved, expressed as percentages.

#### Elevated plus maze

The test was conducted using a plus-cross-shaped apparatus made of grey Plexiglas that was elevated 50 cm above the floor and comprised two open and two closed arms (30 × 7 cm) that extended from a central platform (7 × 7 cm). The Noldus Ethovision 3.0 tracking system was used to record behavior for 5 min. The proportion of entries in the open arms was calculated as the number of entries in the open arms divided by the total number of entries in the closed and open arms, and expressed as a percentage.

#### Tail suspension test

Mice were suspended at approximately one-third from the end of the tail, using regular tape, to a metal rod about 30 cm from the table, for 6 min. Upon viewing of the video recordings blindly to the treatment, the total time spent in an immobile posture was measured and expressed as a percentage.

#### Olfactory preference test

The olfactory preference test was adapted from Pérez-Gomez et al.^21^ and performed in a quiet and dimly lit room. Clean housing cages with regular bedding material were used as testing arenas, covered by transparent Plexiglas lids. A petri dish with a hooled cover was placed and adhered to one side of the arena for defining an odor zone. The Noldus Ethovision 3.0 system was used to track the position and locomotor activity of the mice for 15 min. During the first 4 days, only a Whatman paper filter (GE Healthcare Life Sciences, USA) was placed into the petri dish, to assess the baseline exploration. Then, 2 days were dedicated to each odor with the odorant placed on a paper filter in the following order: peanut oil (pure, 400 µl), female urine (pure, 100 µl), trimethylamine (Sigma-Aldrich, 6.75% in water, 400 µl) and trimethylthiazole (Sigma-Aldrich, 5% in mineral oil, 400 µl). The olfactory preference index was calculated as previously reported^21^, as the difference between the time a given mouse spent in the odor zone when exposed to an odor and the average time of all saline-treated mice in this area during habituation (TH), divided by TH.

#### Olfactory detection test

Mice were trained using a custom-built computer controlled eight-channel olfactometer as previously described^42^ (see also supplemental information). Odorants were diluted in odorless mineral oil (Sigma-Aldrich) for carvone+ (Sigma-Aldrich) or distillated water for 1-butanol (Sigma-Aldrich) to the desired concentration, and 10 ml of solution was used as the odorant source. The pairs of odorants used in the experiments were : carvone+ or 1-butanol *vs* their respective solvants. All mice were first trained to detect an odor at a high concentration without any treatment (10^–2^ v/v for Carvone+ and 10^–3^ v/v for 1-butanol). After initial training, detection threshold concentration were determined in mice injected with saline or GBR. Mice were given at least 200 trials in one session per day, one day out of two. The criterion performance was achieved when the mouse reached ≥ 85% of correct responses in at least one block of 20 trials. The concentration of the odor (S + stimulus) was reduced tenfold in the next session. If criterion performance was not achieved in 300 trials maximum, the preceding concentration was considered as the detection threshold.

#### Gustatory preference test

Mice were individually habituated to two 50 ml bottles filled with drinking water for 24 h. After habituation, the mice were given access to a two-bottle choice of water, or either sucrose solution (1%) or quinine solution (0.1 mM). Bottles containing water and tastant solution were weighed at several time points, 10 a.m., 2 p.m. and 6 p.m., for 48 h with the same solution, starting at 10 AM. The position of the bottles was changed (left to right, right to left) after each weight measurement to ensure that the mice did not develop a side preference. Animal were returned to their home cage for 24 h before starting the test with a new solution. Sucrose preference and quinine aversion were calculated as the percentage (amount of tastant solution consumed × 100/total volume consumed of both tastant and water solution). Water and tastant solution bottles were prepared 24 h before their use and placed in empty cages to check leakage.

### Statistical analysis

Statistical analyses were performed with GraphPad Prism 9 software (USA). Normality was assessed using the Kolmogorov–Smirnov test. The Barlett test was then used to statistically compare the variances. Parametric or non-parametric tests were used accordingly : paired, unpaired or one sample Student, Mann–Whitney or Wilcoxon tests, One-way ANOVA or Kruskal–Wallis tests with repeated measures when suitable, Two-way repeated measures ANOVA or Mixed-effect model when some values were missing. Following post-hoc analyses were applied with Holm–Sidak or Dunn corrections. All animals and data points collected were included in our analysis. The investigators were not blinded during data collection in animal experiments, since only an investigator who gave handling habituation to mice could get access to avoid any types of stress. The investigator knew the group allocation for animal behavior experiments that were automatically quantified by Noldus Ethovision 3.0 (Olfactory preference test, Open field, elevated place maze) or by custom-made automatic olfactometer (olfactory detection test).

All datasets were described using the mean; error bars in the figures represent standard error mean (SEM), except in Fig. 3. Differences were considered significant for p < 0.05.

## Supplementary Information

Supplementary Information.

## Data Availability

The datasets generated and/or analysed during the current study are available in the Mendeley Data repository (http://dx.doi.org/10.17632/7rhz66hwfw.1).
