# Supplementary material for: Assessing positive and negative valence systems to refine animal models of bipolar disorders: the example of GBR 12909-induced manic phenotype
Source: Sci Rep. 2022 May 5;12:7364. doi: 10.1038/s41598-022-10965-8 (PMC9072677; doi:10.1038/s41598-022-10965-8)
Supplement: Supplementary file 1 — Supplementary Information. [file 41598_2022_10965_MOESM1_ESM.pdf]

Supplemental information

Assessing positive and negative valence systems to refine animal models of bipolar disorders: the example of GBR 12909-induced manic phenotype

Supplemental Methods and Materials

Animals

All animal care and experimental procedures followed national and European (2010/63/EU) guidelines and were approved by the French Ministry of Research (APAFiS: #16380-2018080217358599_v1). C57BL/6N male mice (12– 16 weeks old) purchased from Taconic Farms (Denmark) were used for all behavioral tests (n = 74). Once they arrived in the animal facility, the mice were given at least one week to habituate. Mice were socially housed, 4-6 per cage, and maintained under standard housing conditions (23 ± 1 °C; humidity 40%) with a 14/10 h light/dark cycle (lights were on from 6:30 AM to 9:30 PM every day) with food and water *ad libitum*, except for some behavioral experiments. All behavioral tests were conducted during the period of light (10 AM-7 PM). We used the minimum number of animals as estimated from our previous knowledge in performing the same type of experiments.

Behavioral assessment

Olfactory preference test. The test was adapted from Pérez-Gómez et al. (2015). The test was performed in a quiet and dimly lit room (~ 40 lux), around 3 to 6 pm. Clean housing cages (17 x 32 cm) with regular bedding material were used as testing arenas, covered by transparent Plexiglas lids. Each testing arena received mice socially housed in the same cage. The first day, all the mice from the same cage were placed together to overcome neophobia, in each testing arena for habituation during 15-20 min. The second day, a petri dish (94 mm diameter) with a hooled cover was placed and adhered to one side of the arena. For 12 consecutive days, the behavior was recorded by a video camera for 15 min and the Noldus Ethovision 3.0 system (Netherlands) was used to track the position of the mice. The time spent in an odor zone, defined as a rectangle drawn to cover all the cage side around the petri dish and 2 cm from it (representing about one-third of the cage), and the locomotor activity were used as measures

of olfactory valence. During the first 4 days, only a Whatman paper filter (GE Healthcare Life Sciences, USA) was placed into the petri dish, to assess the baseline exploration. Then, 2 days were dedicated to each odor, placed on a paper filter, in the following order: peanut oil (pure, 400 µl), female urine (pure, 100 µl), trimethylamine (Sigma-Aldrich, Germany, 6.75% in water, 400 µl) and trimethylthiazole (Sigma-Aldrich, 5% in mineral oil, 400 µl). Peanut oil and female urine are classical appetitive odorants, whereas trimethylamine and trimethylthiazoline are predator urine synthetic compounds, aversive at these concentrations (Li et al., 2013; Pérez-Gómez et al., 2015; Root et al., 2014). However, in our experimental conditions animals were not food deprived before the test and consequently peanut oil had a neutral value, not triggering attraction. Repeating the measurement of each odor twice allowed to decrease the inter-subject variability of this spontaneous behavior. The olfactory preference index was calculated as explained in Pérez-Gomez et al. (Pérez-Gómez et al., 2015), as the difference between the time a given mouse spent in odor zone when exposed to an odor and the average time of all saline-treated mice in this area when only paper filter was present (TH, during habituation), divided by TH.

Olfactory behavioral testing

Mice were trained using a custom-built computer-controlled eight-channel olfactometer, as previously described (Grelat et al., 2018).

Pretraining Procedures. Partially water-deprived mice (at 80–85 % of their normal body weight) were trained using a go/no-go procedure. First, standard operant conditioning methods were used to train mice to insert their snouts into the odor-sampling port and to respond by retracting the head and licking the water port (located to the left of the odor port). The mice initiated each trial by breaking the light beam positioned across the odor port which led to the opening of an odor valve and a diversion valve directing all airflow away from the sampling tube. This resulted in the odorant vapor being combined with the main air stream and the diversion of the main air stream to an exhaust path. The diversion valve closed 1 s later, and the odor stimulus was presented to the odor sampling port. The stimulus valve closed 2 s later (maximum odor stimulus duration), terminating delivery of the odor. Reward delivery depended on the mouse licking the water delivery tube (maximum response criterion, 2 s). Trials in which the mouse did not keep its snout in the odor-sampling port for at least 0.1 s after odor onset were aborted

and counted as short-sample trials. A 3 μL water reward was delivered if the mouse satisfied the response criterion. The mouse then had to retract its head (the beam was resealed) and wait at least 5 s (intertrial interval) before initiating a new trial. All mice underwent at least five pretraining sessions without odor stimuli. We considered that mice had learned the rules when they responded by introducing their snout in the odor port, waited 1,200 ms before leaving to lick the water port for at least 40 trials, and exhibited fewer than 50 short samples. A pseudorandom protocol was used during behavioral experiments to assign animals to the different olfactometers (six olfactometers in total). A given animal was never trained two consecutive days in the same device.

Training Procedures. The trial procedures were identical to those used in the initial pretraining sessions. Mice were trained to respond to the presence of an odor (S+ : positive stimulus) by licking the water port and to refrain from responding to the presence of odorless mineral oil or water (S− : negative stimulus). In each trial, a single stimulus (S+ or S−) was presented. If the response criterion was met in S+ trials, a droplet of water (3 μL) was given as a reward, and the trial was scored as a hit; if not, the trial was scored as a miss. Failing to lick in a S− trial was scored as a correct rejection (CR); otherwise, the trial was scored as an false alarm (FA). S+ and S− trials were presented in a modified random order (each block contained equal numbers of S+ and S− stimulus trials, and neither stimulus was presented more than three times consecutively). The percentage of correct responses was determined for each block of 20 trials [(hits+CRs)/20×100]. Scores ≥ 85 % implied that mice had correctly learned to assign the reward value to the S+ and the non-reward value to the S−. The odor-sampling time (detection time) was the latency from odor onset to withdrawal of the nose from the odor port. The movement time was the latency from the withdrawal of the nose from the odor port to the first lick on the water port. Detection time and movement time were quantified on Hit trials for the first 5 blocks after reaching the criterion on the highest tested odor dilution.

Resources and Reagents

Chemicals, Peptides, and Recombinant Proteins

GBR 12909	Sigma-Aldrich	D052

Carvone+	Sigma-Aldrich	22070
1-butanol	Sigma-Aldrich	87906
Mineral oil	Sigma-Aldrich	M5904
Peanut oil	Huilerie beaujolaise	
Trimethylamine solution	Sigma-Aldrich	92262
2,4,5-Trimethylthiazole	Sigma-Aldrich	W332518
Sucrose	Sigma-Aldrich	S9378
Quinine hydrochloride dihydrate	Sigma-Aldrich	Q1125
Experimental Models: Organisms/Strains		
Mice: C57BL6/6NTac	Taconic Farms	
**Software and Algorithms**		
Noldus 3.0	EthoVision	https://www.noldus.com/ethovision-xt
Prism	Prism-GraphPad	https://www.graphpad.com/scientific-software/prism/
R	CRAN	https://cran.r-project.org/

Data availability.

The datasets generated and/or analysed during the current study are available in the Mendeley Data repository (http://dx.doi.org/10.17632/7rhz66hwfw.1).

Supplemental Tables and Figures

Group / Odor stimulus	Sal (n = 10)	GBR (on) (n = 9)	GBR (off) (n = 9)
**Peanut oil**	t = 1,26, p = 0,238	t = 0,66, p = 0,525	t = 0,37, p = 0,722
**Female urine**	t = 5,56, ***p < 0,001	t = 0,28, p = 0,783	t = 2,78, *p = 0,024
**TMA**	W = -51,00, **p = 0,006	W = -45,00, **p = 0,004	t = 10,94, ***p < 0,001
**TMT**	W = - 55,00, **p = 0,002	W = -45,00, **p = 0,004	t = 11,79, ***p < 0,001

Table S1 : Attractiveness or aversiveness of olfactory stimuli shown in Figure 3c
The preference index for each odor and group was compared to a theoretical value of 0, by one sample Student or Wilcoxon tests depending on the normality of the data.

Group / Taste stimulus	Sal (n = 17)	GBR (n = 16)
**Sucrose – Day 1**	t = 14,40, ***p < 0,001	t = 2,89, *p = 0,011
**Quinine – Day 4**	t = 4,59, ***p < 0,001	t = 13,80, ***p < 0,001
**Sucrose – Day 2**	t = 3,28, **p = 0,005	t = 4,83, ***p < 0,001
**Quinine – Day 5**	t = 1,28, p = 0,220	t = 13,57, ***p < 0,001

Table S2 : Attractiveness or aversiveness of gustatory stimuli shown in Figure 5c-d
The preference index for each tastant, period and group was compared to a theoretical value of 0, by one sample Student or Wilcoxon tests depending on the normality of the data.

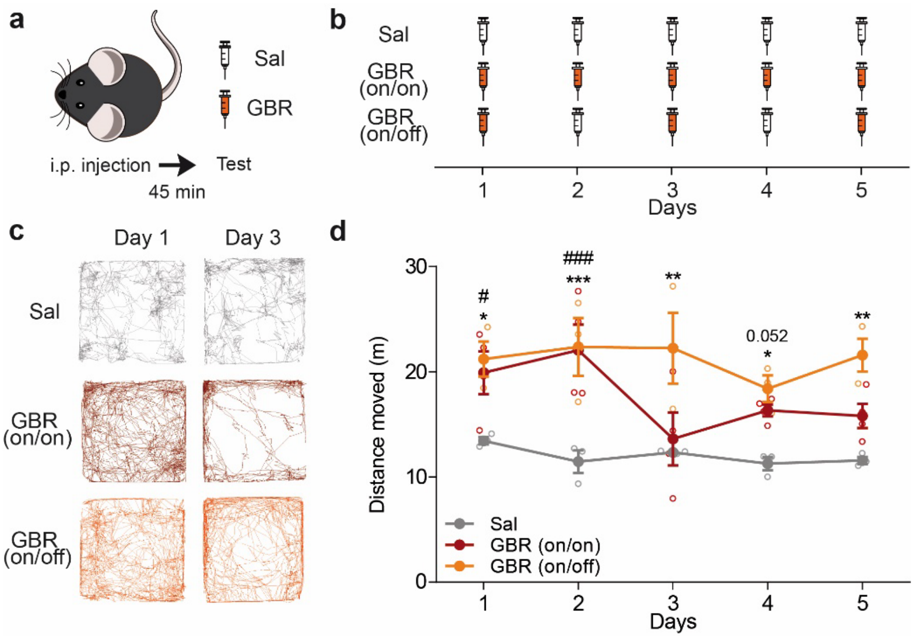

Figure S1 : **Injecting GBR everyday induces tolerance regarding the hyperlocomotion phenotype.** **a**, Either GBR 12909 (GBR) or saline (Sal) was injected intra-peritoneally (i.p.) 45 min before the Open field test (OF). **b**, Timeline for two different injection protocol: GBR (on/on) mice were injected everyday whereas GBR (on/off) received GBR every other day ("on"), alterned with saline ("off") during 5 days. **c**, Representative animal track for the 5 first minutes of the OF. One same animal is represented for Sal, GBR (on/on) and GBR (on/off) groups on days 1 and 3. **d**, OF revealed sustained hyperlocomotion in the GBR (on/off)-treated mice compared with the Sal-treated mice (n = 3) over 5 days, whereas the GBR (on/on) group did not exhibit anymore hyperlocomotion from the day 3 (Two-way repeated measures ANOVA, Group : $F_{(4, 28)} = 3.04$, $p = 0.034$, Days : $F_{(2, 7)} = 11.38$, $p = 0.006$, Interaction : $F_{(8, 28)} = 2.34$, $p = 0.046$ followed by Holm-Sidak post-hoc test : $^{\#}p < 0.05$ and $^{\#\#\#}p < 0.001$, GBR (on/on) *vs* Sal ; $*p < 0.05$, $**p < 0.01$, $***p < 0.001$, GBR (on/off) *vs* Sal, n = 3-4). Data are shown as mean ± SEM and individual data points.

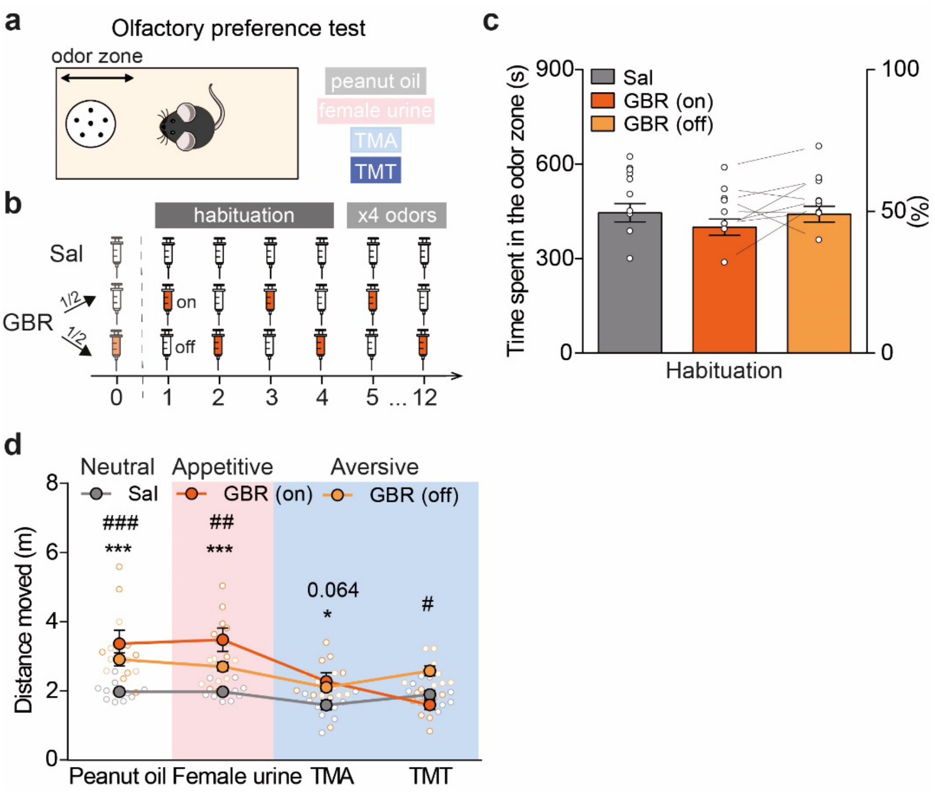

Figure S2 : **The olfactory preference test. a**, Scheme of the olfactory preference test protocol. The test occurred in a standard housing cage, with a covered petri dish installed on one side delimitating the odor zone. After four days of habituation without odorants, each odor was presented on two consecutive days inside the petri dish. Peanut oil and female urine (both pure) are used as neutral and innate attractive odors. TMA (trimethylamine, 6.75 % in water) and TMT (trimethylthiazoline, 5 % in mineral oil) are synthetic predator odors triggering innate aversion. **b**, Timeline of the injections protocol for the olfactory preference test. GBR-treated mice were divided into two groups, altenatively injected each day with either Sal or GBR. **c**, The time spent in the odor zone during the habituation phase of the olfactory preference test is the same across Sal, GBR (on) and GBR (off) mice (Sal *vs* GBR (on) or GBR (off) : One-way ANOVA, $F_{(2, 25)} = 0.87$, $p = 0.432$, n = 9-10 ; GBR (on) *vs* GBR (off) paired Student test, t = 1.42, df = 8, $p = 0.193$, n = 10). **d**, The TMT aversive odor prevented the hyperlocomotion observed during "on", but not during "off" phase after GBR administration (Two-way repeated measures ANOVA, Group : $F_{(2, 25)} = 9.05$, $p = 0.001$, Odor : $F_{(3, 75)} = 25.92$, $p < 0.001$, Interaction : $F_{(6, 75)} = 10.47$, $p < 0.001$ followed by Holm-Sidak post-hoc test : $^{*}p < 0.05$, $^{***}p < 0.001$, GBR (on/off) *vs* Sal ; $^{\#}p < 0.05$, $^{\#\#}p < 0.01$, $^{\#\#\#}p < 0.001$, GBR (off) *vs* Sal, n = 10). Data are shown as mean ± SEM and individual data points.

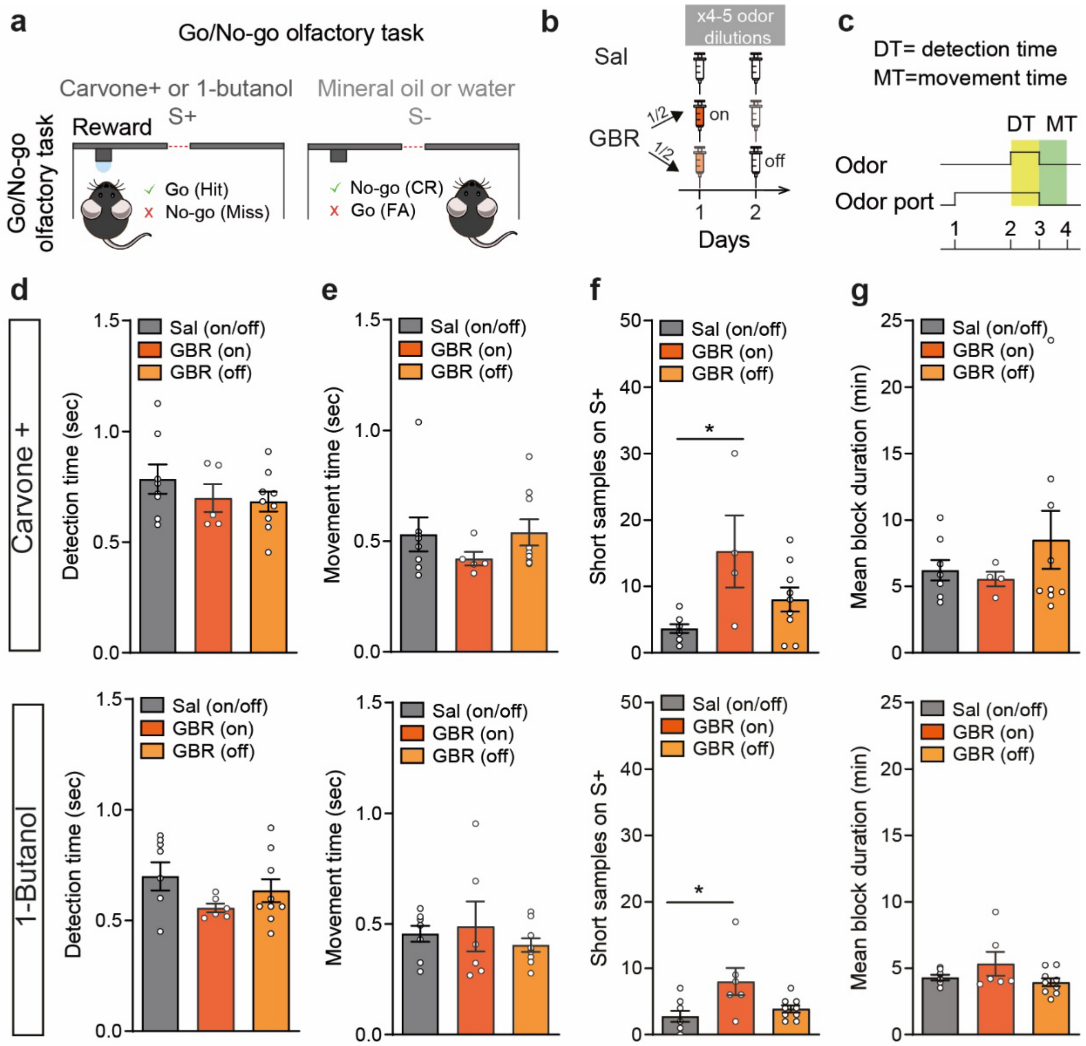

Figure S3. Detection and movement time are not affected by GBR administration. **a**, Schematic of the Go/no-go learning task. In response to S+, licking the water port triggered water delivery. This constitutes a correct response (Go, a hit), whereas not going to lick is considered an error (No-go, a miss). In response to S−, trained mice refrained from licking, thus constituting the correct response (No-go, correct rejection, CR), whereas going to lick constitutes an error (Go, false alarm, FA). **b**, Experimental timeline and injection protocol. All mice were first trained in a detection task without any treatment (carvone + (10^{-2}) *vs* mineral oil; 1-butanol (10^{-3}) *vs* water, data not shown). **c**, Schematic of the times measured in the go/no-go task. (1) The mice initiated each trial by breaking the light beam positioned across the odor sampling port. The odor sampling time (detection time) was the latency from odor onset (2) to withdrawal of the nose from the odor port (3) (yellow shading). The movement time was the latency from nose withdrawal from the odor port (3) to licking of the water port (4) (green shading). **e**, Mean detection time in hit trials for the first five blocks above the criterion for 10^{-2} carvone + dilution (top) and 10^{-3} 1-butanol dilution (bottom). The detection time remained unchanged among groups, supporting the notion that GBR treatment did not change olfactory performance (Kruskal–Wallis test, carvone+ (top): p = 0.647, 1-butanol (bottom): p = 0.311). **e**, Same that in E for the mean

movement time (Kruskal–Wallis test, carvone+ (top): $p = 0.354$; 1-butanol (bottom): $p = 0.703$). **f**, Number of short samples in S+ trials for the first five blocks above the criterion increase in GBR (on) group respect to Sal-injected mice for both tasks (Kruskal–Wallis test, carvone+ (top): $p = 0.032$; 1-butanol (bottom): $p = 0.026$, followed by Dunn's test : $*p < 0.05$) reflecting an increase on impulsivity. **g**, No differerence were found in the mean block duration for the first five blocks above the criterion increase, suggesting no main difference in task engagment or motivation during the tasks (Kruskal–Wallis test, carvone+ (top): $p = 0.953$; 1-butanol (bottom): $p = 0.397$). Data are shown as mean ± SEM and individual data points.

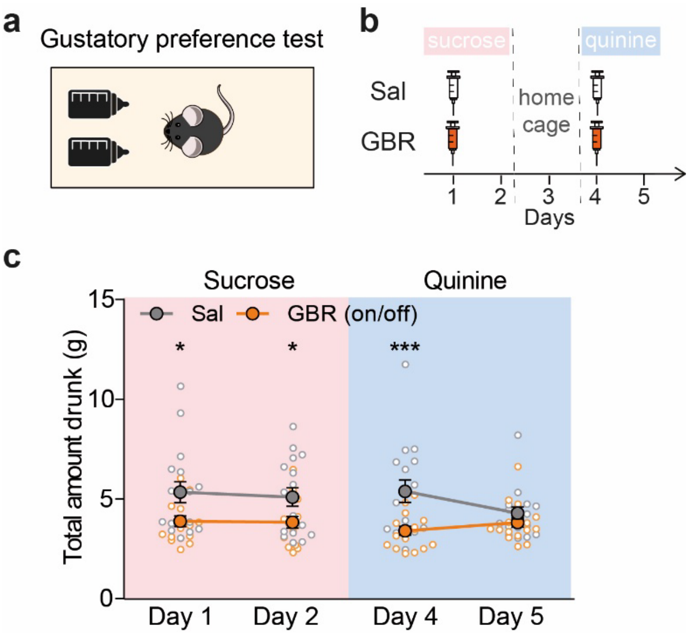

Figure S4 : **The gustatory preference test. a**, Scheme of the gustatory preference test. The test occurred in a standard housing cage, with two bottles containing either water or the tastant solution. After one day of habituation with two water bottles, each tastant solution is presented along with water for 48 h. **b**, Timeline of the injections protocol for the gustatory preference test. Mice were injected at the beginning of the test. **c**, GBR-treated mice drank significantly less solution than Sal-treated mice during the 48 h of sucrose presentation (left) (Two-way repeated measures ANOVA, Group : $F_{(1, 31)} = 5.90$, $p = 0.021$, Period : $F_{(1, 31)} = 1.01$, $p = 0.324$, Interaction : $F_{(1, 31)} = 0.43$, $p = 0.519$, followed by Holm-Sidak post-hoc test : *$p < 0.05$, n = 16-17). For quinine presentation, GBR mice drank significantly less solution only during the first 24h of quinine presentation (right) (Two-way repeated measures ANOVA, Group : $F_{(1, 31)} = 6.42$, $p = 0.017$, Period : $F_{(1, 31)} = 3.32$, $p = 0.078$, Interaction : $F_{(1, 31)} = 16.97$, $p < 0.001$, followed by Holm-Sidak post-hoc test : ***$p < 0.001$, n = 16-17). Data are shown as mean ± SEM and individual data points.

References

Grelat, A., Benoit, L., Wagner, S., Moigneu, C., Lledo, P.-M., & Alonso, M. (2018). Adult-born neurons boost odor–reward association. *Proceedings of the National Academy of Sciences*, *115*(10), 2514-2519. https://doi.org/10.1073/pnas.1716400115

Li, Q., Korzan, W. J., Ferrero, D. M., Chang, R. B., Roy, D. S., Buchi, M., Lemon, J. K., Kaur, A. W., Stowers, L., Fendt, M., & Liberles, S. D. (2013). Synchronous Evolution of an Odor Biosynthesis Pathway and Behavioral Response. *Current Biology*, *23*(1), 11-20. https://doi.org/10.1016/j.cub.2012.10.047

Pérez-Gómez, A., Bleymehl, K., Stein, B., Pyrski, M., Birnbaumer, L., Munger, S. D., Leinders-Zufall, T., Zufall, F., & Chamero, P. (2015). Innate Predator Odor Aversion Driven by Parallel Olfactory Subsystems that Converge in the Ventromedial Hypothalamus. *Current Biology*, *25*(10), 1340-1346. https://doi.org/10.1016/j.cub.2015.03.026

Root, C. M., Denny, C. A., Hen, R., & Axel, R. (2014). The participation of cortical amygdala in innate, odour-driven behaviour. *Nature*, *515*(7526), 269-273. https://doi.org/10.1038/nature13897
